# Diagnostic limitation of laryngostroboscopy in comparison to laryngeal electromyography in synkinesis in unilateral vocal fold paralysis

**DOI:** 10.1007/s00405-021-06714-8

**Published:** 2021-03-10

**Authors:** Isabella Stanisz, Matthias Leonhard, Doris-Maria Denk-Linnert, Berit Schneider-Stickler

**Affiliations:** Department of Otorhinolaryngology, Division of Phoniatrics and Speech Language Therapy, Medical University of Vienna/Vienna General Hospital, Waehringer Guertel 18-20, 1090 Vienna, Austria

**Keywords:** Laryngeal synkinesis, Unilateral vocal fold immobility, Voice diagnostics, Vocal fold immobility, LEMG

## Abstract

**Purpose:**

In clinical practice, laryngo(strobo)scopy (LS) is still mainly used for diagnostics and management of unilateral vocal fold paralysis (UFVP), although only laryngeal electromyography (LEMG) can provide information on causes of vocal fold immobility, especially on possible synkinetic reinnervation after recurrent laryngeal nerve (RLN) injury. The goal of this retrospective study was the evaluation whether signs of synkinetic reinnervation in LS can be objectified in comparison to LEMG data.

**Methods:**

Between 1/2015 and 2/2018, 50 patients with laryngostroboscopically suspected UVFP received routine LEMG examination. The LEMG findings were retrospectively compared with LS findings. The LEMG data analysis focused on the diagnosis of synkinetic reinnervation of the TA/LCA and/or PCA. The digital LS recordings were retrospectively re-evaluated by phoniatricians considering 22 selected laryngostroboscopic parameters.

**Results:**

LEMG revealed synkinesis in 23 (46%) and absence of synkinesis in 27 (54%) patients. None of the 22 parameters showed significant association between patients with synkinetic reinnervation and LS findings. The only laryngostroboscopic parameter that was significantly associated with a silent LEMG signal compared to single fiber activity in LEMG was a length difference on the side of the UVFP (*p*-value 0.0001; OR 14.5 (95% CI 3.047–66.81; Sensitivity 0.5; Specificity 0.9355).

**Conclusion:**

Our findings show that synkinesis cannot be diagnosed using only LS. This study underlines the importance of LEMG in clinical routine for detection of laryngeal synkinesis in patients with UVFP before any further therapeutic steps are initiated to avoid later therapy failure.

## Introduction

Clinical manifestations of unilateral vocal fold paralysis (UVFP) can vary widely. Some patients suffer from severe hoarseness, others have only mild dysphonia. In many patients, symptoms may change over time, even some patients experience voice improvement up to normalization, although the movement of the vocal fold is still impaired. Others report on episodic breathing difficulties several months after the UFVP onset [1], although the glottal width is sufficient. This variability after recurrent laryngeal nerve (RLN) injury has been attributed to synkinetic reinnervation [2]. First mentioned in the literature in the 1980s, laryngeal synkinesis can develop after both unilateral and bilateral RLN injury [2–4].

Currently, the occurrence of synkinetic reinnervation is already known from controlled animal trials [2, 5]. In animal studies of experimental reinnervation, the frequency of synkinesis varies from 66 to 88% [6]. Pitman et al. described reinnervation in a rat model consistently 16 weeks after RLN injury [7]. In human retrospective studies Statham et al. and Maronian et al. reported a similar incidence of synkinesis of about 9% [8, 9]. It is possible that the incidence of synkinesis in humans is widely underestimated due to insufficient workup of impaired vocal fold movement and electromyographical evaluation for synkinesis done too early after onset of UVFP.

Synkinesis in general is induced by misdirected regeneration and aberrant reinnervation. In patients after severe ulnar nerve injury and resuture, patterns of reinnervation and motor unit recruitment showed re-established recruitment of reinnervated motor units, but with abnormal pattern and poor motor function for fine movements [10]. Thomas et al. showed, that irregular recruitment was associated with misdirection of motor axons during regeneration in patients after complete ulnar or median nerve section and resuture. Disorganized recruitment of reinnervated motor units could be caused by misdirected motor axons reinnervating antagonistic muscle fibers with different functions and/or by changes in the activity of the motoneurons [11]. Animal studies even demonstrated that disconnected motor axons do not reinnervate their original muscles specifically [12]. Laryngeal synkinesis per definition results from misdirected reinnervation of adductor axons of the RLN that reinnervate abductor muscles and/or the abductor axons that reinnervate the adductor muscles [2, 13, 14]. Synkinetic reinnervation can also be found when there is a biphasic innervation of the same motor unit endplates by both adductor and abductor motor neurons [15]. It seems that misdirected reinnervation is more frequent than denervation after RLN injury. Crumley stated four types of laryngeal synkinesis, divided into a favorable synkinesis and unfavorable synkinesis with three subtypes [16]. Favorable synkinesis or type I synkinesis describes impairment of vocal fold movement without or little phonatory or airway problems. Unfavorable type II-IV synkinesis characterizes vocal fold motion impairment and phonatory and/or airway deficiency. Diagnostic classification into these four subtypes of synkinesis is primarily based on LS and additionally on LEMG. In patients with favorable type I synkinesis no treatment is recommended. In type II synkinesis with involuntary vocal fold movements and jerks, the local application of botulinum toxin (Botox©) is the short-term therapy of choice. As a long-acting treatment option, Ansa cervicalis reinnervation is proposed, which presumably leads to a favorable type of synkinesis. Patients with type III synkinesis present with a medialized vocal fold and vocal process and intermediate to normal voice quality with or without possible airway deficit. Once more Botox© application is recommended for short term treatment. A surgical approach, consisting of repositioning of the arytenoid, partial laser arytenoidectomy or cordotomy, is suggested for long term treatment. Type IV synkinesis in an explicit form is characterized by vocal fold abduction during phonation resulting in a very breathy voice, without airway impairment. Arytenoid adduction or reinnervation is advocated for treatment in type IV synkinesis [16]. In conclusion, therapy recommendations are based on the patient’s symptoms and different synkinesis types, which are primarily observed by LS and to a lesser degree are confirmed with LEMG.

According to Crumley, an understanding of synkinesis, partial regeneration, adductor-abductor competition, and denervation atrophy is prerequisite for successful treatment of patients with RLN injuries [16]. To provide the best therapy for the patient, laryngeal synkinesis must be diagnosed accurately. In clinical practice, the diagnosis of UVFP has mainly relied on LS. Nevertheless, so far LEMG has still been rarely used in diagnostics of UVFP [17, 18]. In the USA and Western Europe only a quite low percentage of the otolaryngologists, 1.7% and 3.6%, respectively, use LEMG for diagnostics and estimating prognosis in UVFP/bilateral vocal fold paralysis (BVFP) [17, 18]. Arguments in favor of LS are easier feasibility, availability, and less discomfort for the patient during the examination compared to LEMG. Since 72% of laryngologists in the USA and 57.1% in Western Europe base their diagnosis of UVFP on LS [17, 18], the question arises, if synkinesis can be correctly diagnosed with laryngostroboscopic examination.

The goal of this retrospective study was the evaluation whether synkinetic reinnervation in UVFP can be objectified by LS compared to LEMG.

## Materials and methods

### Study design and subjects

Patients with laryngostroboscopically suspected UVFP, who underwent LEMG at least 2 months after UVFP onset, were included in this study. Patients were selected after a retrospective chart review of 82 patients, who received LEMG due to laryngostroboscopically diagnosed UVFP at the Division of Phoniatrics and Speech Language Therapy at the Department of Otolaryngology – Head and Neck Surgery, Medical University of Vienna, Austria, between 1/2015 and 2/2018. The study protocol was approved by the Ethics Committee of the Medical University of Vienna (#1175/2018).

Only patients who had undergone diagnostic LEMG and LS after at least 2 months after onset of symptoms for UVFP were included in this retrospective study. The timing of the LEMG investigation is in concordance with the findings of Pitman et al., who could show that synkinesis can be expected earliest 8 weeks and reinnervation seems to be mature 16 weeks after RLN injury [7]. Patients with UVFP onset less than 2 months were excluded. LEMG excluded cricoarytenoid ankylosis and confirmed UVFP in all cases.

### Laryngostroboscopy (LS)

LS was routinely performed prior to LEMG by an otolaryngologist/phoniatrician. A standard vocabulary for description of UVFP does not exist yet, and therefore, laryngostroboscopic criteria were selected from literature review upon the diagnostics of UVFP[19–21]. For study purpose, 22 laryngostroboscopic parameters were retrospectively evaluated by two experienced raters (IS and BSS) by re-judging of the recordings using qualitative feature classification:

- Glottal gap (I/1 = complete closure; I/2 = incomplete closure in the cartilaginous part; I/3 = triangular incomplete closure anterior to the vocal process; I/4 = triangular incomplete closure of the posterior third of the vocal folds; I/5 = incomplete closure of the posterior two thirds; I/6 = incomplete closure all along the folds with posterior gap; IIA/7 = spindle shaped incomplete closure; hour glass shape) [22],
- vocal fold (VF) length difference (no/ yes/ not detectable),
- height difference of VF (no/ yes/ not detectable),
- supraglottic activity (no; anterior/posterior; latero-medial bilateral; latero-medial unaffected side; lateromedial affected side; circumferent; not detectable),
- motility of the arytenoid cartilage without VF motility (no/yes/not detectable),
- irregularities in laryngostroboscopy (no/ yes/ not detectable),
- phase differences (no/ yes/ not detectable),
- position of paretic VF in respiration (median/ paramedian/ intermediate/ lateral/ not detectable),
- adduction of VF at the side of UVFP (midline/ decreased/ none/ not detectable),
- abduction of VF at the side of UVFP (midline/ decreased/ none/ not detectable),
- excavation of VF at the side of UVFP (no/ yes/ not detectable),
- mucosal wave at the side of UVFP (normal/ enlarged/ decreased/ not detectable),
- amplitude of VF at the side of UVFP (normal/ enlarged/ decreased/ not detectable),
- arytenoid cartilage position at the side of UVFP (upright positioned/ collapsed to midline without affecting vocal fold closure/ collapsed to midline with affecting vocal fold closure/ not detectable),
- arytenoid cartilage outward rotation during phonation at the side of UVFP (no/ yes/ not detectable)
- Overlapping of arytenoids (no/ yes),
- and adduction (midline/ overcompensating/ none/ not detectable) of the unaffected VF,
- abduction of the unaffected VF (normal lateral/ decreased/ none/ not detectable), excavation of the unaffected VF (no/ yes/ not detectable),
- mucosal wave of the unaffected VF (normal/ enlarged/ decreased/ not detectable),
- amplitude of the unaffected VF (normal/ enlarged/ decreased/ not detectable) and
- other pathologies (no/ yes).

### Laryngeal electromyography (LEMG)

The LEMG was performed by two otolaryngologists/phoniatricians (ML and BSS). Concentric disposable needle electrodes with a recording area of 0.07 mm^2^ (TECA Elite, Care Fusion, Middleton, Wisconsin, USA) were used for LEMG examination. As electromyography device a Nicolet VikingQuest electromyography acquisition device (Natus Medical Incorporated, Pleasantin, CA, USA) was used.

Patients were awake and not sedated during the examination. Some patients received a cough medication containing dihydrocodeine prior to the examination. For local anesthesia about 1 mL of 1% lidocaine with 1:100,000 dilution of epinephrine was injected subcutaneously in the skin over the cricothyroid ligament. An unidirectional microphone was placed about 20 cm from the patient’s mouth and the voice was recorded time synchronous with LEMG signal. Since Volk et al. mentioned that, the thyroarytenoid muscle (TA) and lateral cricoarytenoid muscle (LCA) are nearly on the same axis and in direct contact to each other. It is thus not always possible to reliably differentiate between these two muscles [23]. This is the reason the term TA/LCA complex was used in this study. Following the LEMG protocol, the following muscles were routinely tested by needle electrode insertion: TA/LCA complex, posterior cricoarytenoid muscle (PCA), cricothyroid muscle (CT). Test maneuvers for agonistic activity were inspiration for CT, phonation for TA /LCA complex and sniffing for PCA testing. Antagonistic intentional activity was tested during sniffing for TA/LCA complex and phonation for PCA testing.

The LEMG signal from the tested muscles were analyzed and categorized for intentional activity into silent, single fiber activity, decreased, dense, spontaneous activity, not performed (NP), not found (ND) and synkinesis (yes/no). LEMG signals density was not considered in this study. Synkinesis was diagnosed in patients with detectable motor unit activation potentials (MUAPS) in agonistic and antagonistic maneuvers in TA/LCA complex and/or PCA muscles, as suggested by Maronian et al.[8]. Diagnosis of synkinesis was made, at a rate of minimum 30% activity during an intentional antagonistic activation compared to agonistic activation. Data from LS and LEMG examination of the same day or the examinations that were least apart from each other were used for analysis.

### Statistical analysis

Descriptive statistics were used to evaluate the demographic data. Statistical analysis was performed using SPSS software (version 21.0; IBM SPSS Inc., IL, USA, RRID:SCR_019096) and GraphPad Prism (Version 7.03, GraphPad Software Inc., CA, USA, RRID:SCR_002798). The association of clinical findings in LS and the presence of synkinesis were analyzed by Fisher’s exact test. Methods used to compute CIs were Baptista Pike for Odds ratios and Wilson Brown for Sensitivity and Specificity. Association of LS findings and LEMG signal was tested by a multivariate log regression model. A *p*-value of < 0.05 was considered statistically significant.

## Results

### Clinical parameters

Fifty patients receiving LEMG were included in this study with laryngostroboscopically suspected UVFP of over 2 month symptom duration. The demographic and clinical data of the 50 patients enrolled in this study are described in Table 1.

**Table 1:** Demographic data of patients (*n* = 50)

Clinical parameter		Values (%)
Gender	Male	25 (50)
	Female	25 (50)
Age at diagnosis (years)	Mean (range)	57 (27–86)
Affected vocal fold	Left	37 (74)
	Right	13 (26)
Etiology	Iatrogenic	36 (72)
	Idiopathic	11 (22)
	Neurogenic	2 (4)
	Tumorous	1 (2)

The main etiology of UVFP was iatrogenic (70.5%), of which 15 patients (38.89%) had thyroid surgery, 13 (33.33%) thoracic surgery, 9 (25%) neck surgery and one (2.78%) surgical resection of an ependymoma in the fourth ventricle.

### LEMG findings

Median time point of LEMG examination was 5 months after symptom onset (range: 2–120 months). LEMG revealed synkinesis in 23 (46%) of UVFP patients and absence of synkinesis in 27 (54%) of patients. The synkinesis group was significantly associated with a higher probability of a surgical etiology when compared with the non-synkinesis group (p = 0.0204; OR 6.967; 95% CI 1.421–33.96; Sensitivity 0.9048; Specificity 0.4231).

### Laryngostroboscopic findings

The following findings were described;

- Position of the paretic vocal fold was median in 6 (12%), paramedian in 34 (68%), intermediate in 7 (14%) and lateral in 3 (6%) patients.
- Glottal gap showed the following results: complete closure was observed in 9 (18%) patients, incomplete closure in the cartilaginous part in 5 (10%) patients, triangular incomplete closure anterior to the vocal process in 2 (4%) patients, triangular incomplete closure of the posterior third of the vocal folds in 6 (12%) patients, incomplete closure of the posterior two third in 7 (14%) patients, incomplete closure all along the folds with posterior gap in 16 (32%) patients and spindle shaped incomplete closure or hour glass shape was found in 5 (10%) patients.
- Adduction on the affected side of UVFP was classified into no adduction, decreased adduction, and adduction to midline in 24 (48%), 22 (44%), and 4 (8%) patients, respectively.
- Decreased abduction on the side of the UVFP was observed in 6 (12%) patients, whereas no movement was found in 44 (88%) patients.
- Excavation of the affected side: Seventeen (34%) patients had an excavated paretic vocal fold and 33 (66%) had no excavation.
- Supraglottic activity was observed as follows: no supraglottic activity, latero-medial bilateral, latero-medial on the healthy side and circumferent supraglottic closure in 20 (40%), 8 (16%), 18 (36%), 4 (8%) patients, respectively.
- The position of the arytenoid cartilage was found to be an upright position on the side of the UVFP in LS in 22 (43.14%) patients, collapsed to midline without affecting vocal closure in 22 (43.14%) patients and collapsed to midline with affecting vocal closure in 6 ( 11.76%) patients. An arytenoid outward rotation during phonation was detected in 3 (5.88%) patients and an “Arytenoid overlapping phenomenon” noted in 4 (7.84%) patients.

### Laryngeal synkinesis and laryngostroboscopic findings

The results of the multivariate log regression model showed no significant association between synkinetic reinnervation and LS findings. The results are listed in Table 2.

**Table 2:** Results of laryngostroboscopic (parameters total number and significance level) for synkinetic and nonsynkinetic reinnervation in unilateral vocal fold immobility diagnosed with laryngeal electromyography

Laryngostroboscopic parameters	*p*-value		Synkinesis (*n*)	No Synkinesis (*n*)
Glottal gap	0.471	I/1 = complete closure	6	3
		I/2 = incomplete closure in the cartilaginous part	1	4
		I/3 = triangular incomplete closure anterior to the vocal process	1	1
		I/4 = triangular incomplete closure of the posterior third of the vocal folds	2	4
		I/5 = incomplete closure of the posterior two thirds	3	4
		I/6 = incomplete closure all along the folds with posterior gap	7	9
		IIA/7 = spindle shaped incomplete closure or hourglass shape	3	2
Vocal fold length difference	0.141	No	20	20
		Yes	3	7
		Not detectable	22	26
Height difference of vocal folds	0.956	Yes	1	0
		Not detectable	0	1
Supraglottal activity	0.798	No	8	11
		Anterior/posterior	0	0
		Latero-medial bilateral	3	4
		Latero-medial healthy	10	9
		Lateromedial affected side	0	0
		Circumferential	2	2
		Not detectable	0	0
Motility of the arytenoid cartilage without VF mobility	0.764	No	21	22
		yes	2	4
Irregularities in LS	0.435	No	2	3
		Yes	3	6
		Not detectable	18	17
Phase differences	0.378	No	1	0
		Yes	3	9
		Not detectable	19	17
Position of the paretic VF in respiration	0.557	Median	4	2
		Paramedian	15	19
		Intermediate	3	4
		Lateral	1	1
		Not detectable	0	0
Adduction on the side of UVFP	0.445	Midline	2	2
		Decreased	9	14
		None	12	10
		Not detectable	0	0
Abduction on the side of UVFP	0.539	Midline	0	0
		Decreased	2	4
		None	21	22
		Not detectable	0	0
Excavation on the side of UVFP	0.331	No	14	20
		Yes	9	6
		Not detectable	0	0
Mucosal wave on the side of UVFP	0.284	Normal	3	2
		Enlarged	1	0
		Decreased	0	2
		Not detectable	19	22
Amplitude on the side of UVFP	0.351	Normal	3	2
		Enlarged	1	1
		Decreased	0	2
		Not detectable	19	21
Arytenoid cartilage position on the side of UVFP	0.126	Upright positioned	15	2
		Collapsed to midline without affecting vocal fold closure	6	16
		Collapsed to midline with affecting vocal fold closure	2	2
		Not detectable	0	1
Arytenoid cartilage outward rotation during phonation	0.060	No	23	23
		Yes	0	3
		Not detectable	0	0
Overlapping of arytenoids	0.096	No	23	23
		Yes	0	3

*VF* vocal fold, *UVFP* unilateral vocal fold paresis

There was no association between the time after onset of UVFP and an upright position of the arytenoid cartilage compared to a collapsed arytenoid cartilage (*p*-value 0.9051; OR 0.9375 (95% Confidence Interval (CI) 0.3193–2.654)); Sensitivity 0.7143; Specificity 0.2727). Position of the paretic vocal fold (*p*-value 0.4006; OR 0.2843; 95% CI 0.02354 – 2.141; Sensitivity 0.055; Specificity 0.8286), height difference (*p*-value 0.54559; OR 0.55, 95% CI 0.04054–3.943; Sensitivity 0.048; Specificity 0.9167), supraglottic activity (*p*-value 0.4203; OR 1.653; 95% CI 0.5781–4.566; Sensitivity 0.6364; Specificity 0.4857) and position of the arytenoid cartilage (*p*-value 0.4998; OR 0.5; 95% CI 0.1436–1.976; Sensitivity 0.333; Specificity 0.5) cannot predict LEMG findings concerning single fiber activity or no activity. The only parameter that was significantly associated with a silent vocal fold compared to single fiber activity in LEMG was a length difference on the side of the UVFP (*p*-value 0.0001; OR 14.5 (95% CI 3.047–66.81; Sensitivity 0.5; Specificity 0.9355).

## Discussion

Synkinetic reinnervation is an important differential diagnosis in unilateral vocal fold immobility. Goal of this study was the evaluation, whether laryngeal synkinesis can be diagnosed reliably by sole LS or only in combination with LEMG. LEMG is still rarely used in otorhinolaryngology yet, but, meanwhile, widely accepted as an excellent diagnostic tool to confirm and determine prognosis in patients with UVFP [24]. Nevertheless, most patients with vocal fold immobility are still examined preferably by LS. As reported in previous studies, LEMG findings of synkinesis are important due to the fact that synkinesis is often associated with a reduced likelihood of recovery of purposeful vocal fold motion and may be a plausible explanation for treatment failure [9]. Management of laryngeal synkinesis was proposed by Crumley, depending on the different type of synkinesis [16]. Crumley defined four subtypes of synkinesis:

- “Favorable synkinesis” as type I synkinesis with partial or complete motion impairment, but with little or no phonatory or airway deficit or disturbance.
- “Unfavorable synkinesis”, of which there are 3 subtypes, consists of abnormal innervation of intrinsic muscles with motion impairment and phonatory and/or airway deficit or disturbance. The three subclasses are as followed: spasms, jerking, or twitching of vocal folds, arytenoids, or false vocal folds (type II); hyperadduction (type III); and hyperabduction (type IV).

The four classes of synkinesis according to Crumley, depicted herein, help explain the clinically different symptoms in UFVP and provide a physiological basis for the treatment of each type [16].

Thyroplasty type I, partial laser arytenoidectomy or cordotomy, surgical repositioning of the arytenoid, vocal fold augmentation and botulinum toxin (Botox©) injections have been suggested as treatment options for UVFP and presence of a certain subtype of laryngeal synkinesis [16, 25]. As already recommended by Crumley, an ansa cervicalis-RLN anastomosis for nonselective laryngeal reinnervation should be considered as “unfavorable synkinesis” to turn it into a “favorable” one.

Maragos analyzed voice outcomes in patients after revision thyroplasty due to the patient's desire to have a better voice or less airway resistance after the initial procedure. The results of the study described that 80% of patients subjectively improved after revision thyroplasty [26]. Yet, there are treatment failures in patients after laryngeal framework surgery despite revision surgery. These treatment failures suggest the assumption that synkinetic reinnervation may be a reason. Hence, synkinesis should be identified before initiation of any treatment so that all potential treatment options can be considered, at least in case of treatment failure. The high percentage of 46% of laryngeal synkinesis in this study in cases of UVFP is comparable to the literature, although the reported incidence can vary. Lin et al. described adductor synkinesis in 30% of patients with chronic vocal fold paresis in a retrospective study [27], whereas Statham et al. reported an incidence of synkinesis of 9.7% and Maronian et al. an incidence of 2% [8, 9]. Statham et al. performed LEMG between 4 weeks and 6 months from the time of diagnosis, whereas Lin et al. performed LEMG at least 6 months after onset of UVFP [9]. Maronian et al. routinely evaluated patients who had vocal fold immobility longer than 12 months by LEMG and identified 9.09% patients with synkinesis [8]. Blitzer et al. found that 50% of patients with good voice performance despite vocal fold immobility had evidence of synkinetic reinnervation on LEMG [5]. The inconsistently stated incidence of laryngeal synkinesis may be due to different timing of LEMG after diagnosis of vocal fold paresis. On one hand, patients possibly were evaluated too early after onset of UVFP and laryngeal reinnervation, and therefore, synkinesis may not yet be present [7]. Timing of LEMG should, therefore, be considered depending on the underlying pathology of UVFP. In this study only patients who underwent LEMG later than 2 months after onset of symptoms were included. Furthermore, if LEMG is not performed routinely after UVFP, patients with interim voice improvement are not evaluated by LEMG, and synkinesis could be overlooked. Voice improvement may be due to synkinetic reinnervation. LEMG is crucial for diagnostics of synkinetic reinnervation and cannot be replaced by LS [5, 28]. Moreover, routine LEMG of patients with UVFP has to be performed to estimate the real incidence of laryngeal synkinesis.

Statham et al. defined synkinesis as abnormal TA-LCA muscle contraction during abductor tasks in patients with aberrant vocal fold movement for less than 6 months [9]. However, there is still no consent on uniform diagnostics criteria for laryngeal synkinesis in the literature. Maronian et al. proposed the following definition of synkinesis in LEMG, which also was used in this study: Synkinesis of the TA/LCA complex was defined as muscle activity during sniffing that was greater or equal to muscle activity during phonation, PCA synkinesis was defined as significant muscle activity during phonation [8].

This study compared LS findings with LEMG data. In detail, we correlated twenty-two LS parameters with LEMG findings to identify LS parameters that could predict synkinesis in our patient group. In our study none of the twenty-two above-mentioned LS findings had a statistically significant potential to diagnose synkinesis as seen in LEMG. Only length difference of the paretic vocal fold was significantly associated with a silent LEMG finding compared to single fiber activity. Woodsen et al. described that a paralyzed vocal fold tends to be shortened, with anterior rotation of the arytenoid [21]. Lin et al. hypothesized that synkinesis likely maintain laryngeal muscle tone, resulting in medialization of the paretic vocal fold and eventually explaining a better voice outcome in patients with synkinesis [28]. Our data did not suggest an association between the position of the vocal fold and synkinesis in general (*p* = 0.4006).

In clinical practice, although most otolaryngologists perform LS for diagnostics of UVFP, synkinetic reinnervation might be overseen by considering only LS [17, 18]. Arguments supporting LS are easier feasibility, availability, and less discomfort for the patient during the examination compared to LEMG. Nevertheless, in summary synkinetic reinnervation can have therapeutic impact and can only be diagnosed by LEMG and not exclusively by LS. Thus, LEMG has to be part of the standard examination procedures and should be implemented not only in neurolaryngology specialized centers but also in daily routine practice.

In conclusion, our findings underline the importance of LEMG in diagnosis of laryngeal synkinesis in patients with UVFP.

## Data Availability

Data and material are available upon request.
